# Supplementary material for: HIVprotI: an integrated web based platform for prediction and design of HIV proteins inhibitors
Source: J Cheminform. 2018 Mar 9;10:12. doi: 10.1186/s13321-018-0266-y (PMC5845081; doi:10.1186/s13321-018-0266-y)
Supplement: Supplementary file 2 — Additional file 2. Source code of HIVProtI web server. [file 13321_2018_266_MOESM2_ESM.zip › HIVprotI_Source-code/hivprotI/weka-3-6-6/doc/weka/experiment/RemoteEngine.html]

RemoteEngine


---


|  |  |  |
| --- | --- | --- |
| **Overview** \| **Package** \| **Class** \| **Tree** \| **Deprecated** \| **Index** \| **Help** \| **Weka's home** | |  |
| **PREV CLASS**   **NEXT CLASS** | **FRAMES**    **NO FRAMES**     **All Classes** |  |
| SUMMARY: NESTED \| FIELD \| CONSTR \| METHOD | DETAIL: FIELD \| CONSTR \| METHOD |  |


---


## weka.experiment Class RemoteEngine

```
java.lang.Object
  java.rmi.server.RemoteObject
      java.rmi.server.RemoteServer
          java.rmi.server.UnicastRemoteObject
              weka.experiment.RemoteEngine
```

**All Implemented Interfaces:**: java.io.Serializable, java.rmi.Remote, RevisionHandler, Compute

---

``` public class RemoteEngine extends java.rmi.server.UnicastRemoteObject implements Compute, RevisionHandler ```

A general purpose server for executing Task objects sent via RMI.

**Version:**
:   $Revision: 1.12 $

**Author:**
:   Mark Hall (mhall@cs.waikato.ac.nz)

**See Also:**: Serialized Form

---

**Constructor Summary**

`RemoteEngine(java.lang.String hostName)`
  
          Constructor


| **Method Summary** | |
| --- | --- |
| `java.lang.Object` | `checkStatus(java.lang.Object taskId)`             Returns status information on a particular task |
| `java.lang.Object` | `executeTask(Task t)`             Takes a task object and queues it for execution |
| `java.lang.String` | `getRevision()`             Returns the revision string. |
| `static void` | `main(java.lang.String[] args)`             Main method. |

**Methods inherited from class java.rmi.server.UnicastRemoteObject**

`clone, exportObject, exportObject, exportObject, unexportObject`

**Methods inherited from class java.rmi.server.RemoteServer**

`getClientHost, getLog, setLog`

**Methods inherited from class java.rmi.server.RemoteObject**

`equals, getRef, hashCode, toString, toStub`

**Methods inherited from class java.lang.Object**

`getClass, notify, notifyAll, wait, wait, wait`

**Constructor Detail**

### RemoteEngine

```
public RemoteEngine(java.lang.String hostName)
             throws java.rmi.RemoteException
```

:   Constructor

    **Parameters:**: `hostName` - name of the host **Throws:**: `java.rmi.RemoteException` - if something goes wrong


**Method Detail**

### executeTask

```
public java.lang.Object executeTask(Task t)
                             throws java.rmi.RemoteException
```

:   Takes a task object and queues it for execution

    :   **Specified by:**: `executeTask` in interface `Compute`
    :   **Parameters:**: `t` - the Task object to execute **Returns:**: an identifier for the Task that can be used when querying Task status **Throws:**: `java.rmi.RemoteException` - if something goes wrong.

---


### checkStatus

```
public java.lang.Object checkStatus(java.lang.Object taskId)
                             throws java.lang.Exception
```

:   Returns status information on a particular task

    :   **Specified by:**: `checkStatus` in interface `Compute`
    :   **Parameters:**: `taskId` - the ID of the task to check **Returns:**: a `TaskStatusInfo` encapsulating task status info **Throws:**: `java.lang.Exception` - if an error occurs

---


### getRevision

```
public java.lang.String getRevision()
```

:   Returns the revision string.

    :   **Specified by:**: `getRevision` in interface `RevisionHandler`
    :   **Returns:**: the revision

---


### main

```
public static void main(java.lang.String[] args)
```

:   Main method. Gets address of the local host, creates a remote engine
    object and binds it in the RMI registry. If there is no RMI registry,
    then it tries to create one with default port 1099.

    :   **Parameters:**: `args` -


---


|  |  |  |
| --- | --- | --- |
| **Overview** \| **Package** \| **Class** \| **Tree** \| **Deprecated** \| **Index** \| **Help** \| **Weka's home** | |  |
| **PREV CLASS**   **NEXT CLASS** | **FRAMES**    **NO FRAMES**     **All Classes** |  |
| SUMMARY: NESTED \| FIELD \| CONSTR \| METHOD | DETAIL: FIELD \| CONSTR \| METHOD |  |


---
